# Oral Rehydration Therapy in the Second Decade of the Twenty-first Century

**DOI:** 10.1007/s11894-014-0376-2

**Published:** 2014-02-22

**Authors:** Henry J. Binder, Ian Brown, B. S. Ramakrishna, Graeme P. Young

**Affiliations:** 1 Department of Internal Medicine, Yale School of Medicine, P.O. Box 208019, New Haven, CT 06520 USA; 2 Flinders Centre for Innovation in Cancer, Flinders University, Adelaide, SA Australia; 3 SRM Institutes for Medical Sciences, Vadapalani, Chennai, 600 026 India

**Keywords:** Acute diarrhea, Dual-action oral rehydration solution, Fermentable/resistant starch, Oral rehydration solution, Short-chain fatty acids

## Abstract

Oral rehydration solution (ORS) was established as the cornerstone of therapy for dehydration secondary to acute infectious diarrhea approximately 40 years ago. The efficacy of ORS is based on the ability of glucose to stimulate Na and fluid absorption in the small intestine via a cyclic AMP-independent process. Despite the establishment that ORS is the primary reason for the substantial reduction in morbidity and mortality from diarrhea in children in developing countries, the use of ORS has lagged for many reasons. This review highlights efforts to establish a major reformulation of ORS following the demonstration that short-chain fatty acids (SCFA) stimulate colonic Na and fluid absorption by a cyclic AMP-independent mechanism. The addition of high-amylose maize starch (HAMS), a microbially-fermentable (or ‘resistant’) starch, to ORS results in delivery of non-absorbed carbohydrate to the colon where it is fermented to SCFA. To date, three randomized controlled trials with a HAMS-ORS in south India have demonstrated a substantial decrease in diarrhea duration in both adults and children hospitalized for acute diarrhea. Significant efforts are now underway to establish this dual-action, modified HAMS-hypoosmolar ORS solution as the standard ORS for the treatment of dehydration from acute diarrhea.

## Introduction

Oral rehydration solution (ORS) was originally developed in the early 1970s to correct the substantial dehydration that occurs as a result of severe diarrhea, especially acute infectious diarrhea [1•, 2•, 3]. Though rehydration with intravenous (IV) solutions has been employed for over a century, the logistics of IV hydration with the need for sterile solutions are monumental when hundreds and possibly thousands of individuals are affected by large volume diarrhea (e.g., cholera) in developing countries in field conditions with minimal medical resources, including elemental sanitation and electricity. In its simplest and original form, ORS was an iso-osmolar, glucose-electrolyte solution with added base (e.g., citrate in WHO-ORS) that was designed to correct dehydration and metabolic acidosis [3].

## Development of ORS

Three unrelated events during a 10-year period in 1960s and 1970s led to the development and employment of ORS in the treatment of acute diarrhea. The initial scientific observations that were critical to the subsequent development of ORS were the establishment that glucose absorption in the mammalian small intestine required luminal Na, and that Na absorption was markedly enhanced by the presence of luminal glucose (as well as luminal amino acids) [4]. Schultz and Zalusky provided evidence for glucose-stimulated Na absorption and Na-dependent glucose absorption in a series of experiments with in vitro rabbit ileum [5•]. Subsequent studies identified the intestinal glucose-Na transporter as SGLT1 which has been extensively studied by Wright and colleagues [6].

**Fig. 1:**
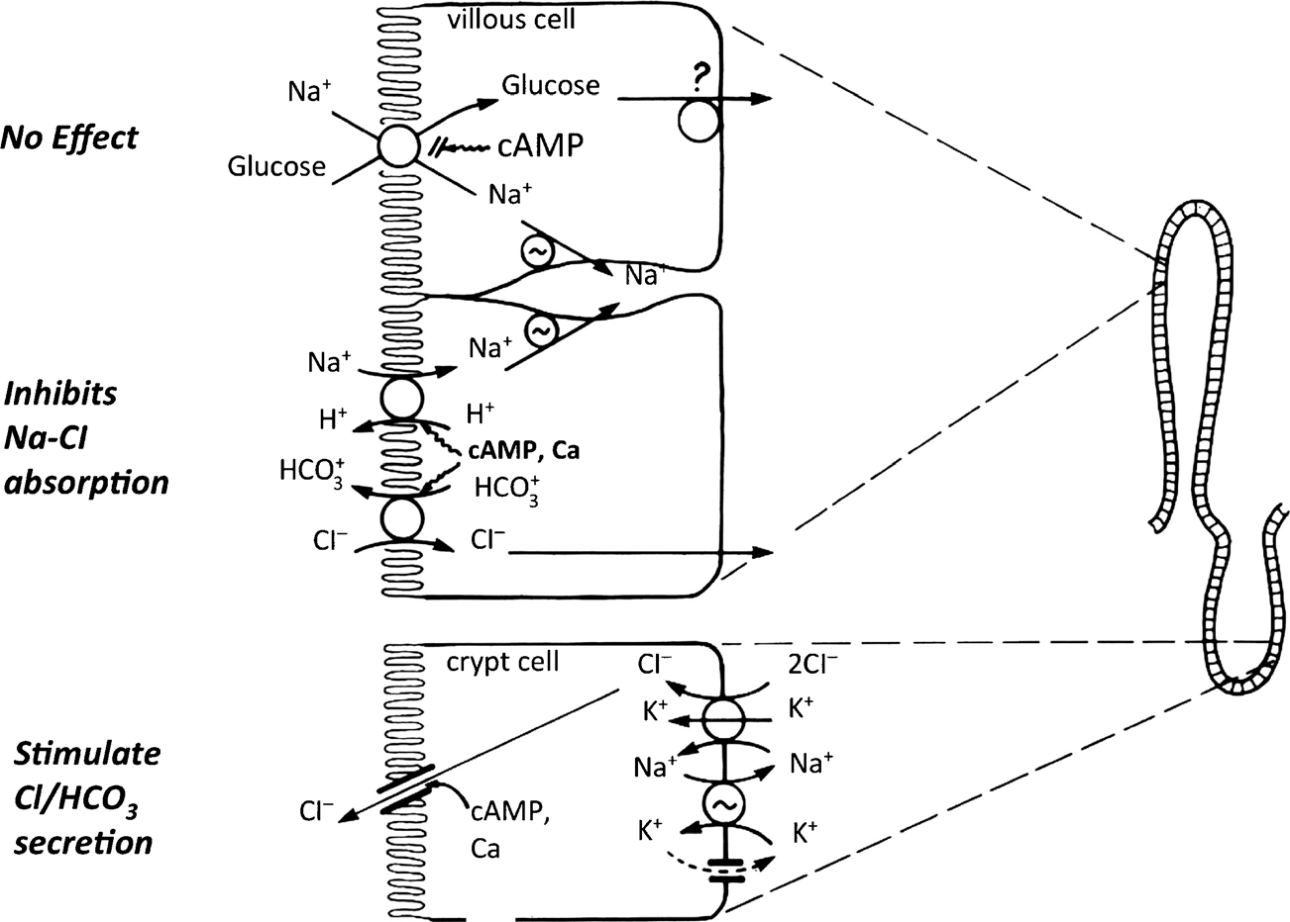
Physiologic basis of efficacy of oral rehydration solution (ORS): electrolyte absorption occurs in villous cells, while secretion in crypt cells. Increases in intracellular cAMP inhibit Na-Cl absorption (that is the result of parallel Na-H and Cl-HCO_3_ exchanges) in villous cells and stimulates active Cl and/or HCO_3_ secretion in crypt cells. In contrast, glucose-stimulated Na absorption also in villous cells is not affected by increases in intracellular cAMP. Thus, the glucose enhances Na and fluid absorption despite persistence of stimulation of Cl secretion and inhibition of Na-Cl absorption

The second critical observation was the result of several studies that established that: (1) cholera enterotoxin caused diarrhea in clinical cholera by inducing fluid and electrolyte secretion via activation of adenylate cyclase resulting in an increase in cyclic AMP in intestinal epithelial cells [7]; and (2) the action of cyclic AMP on intestinal epithelial transport included both stimulation of active Cl secretion and inhibition of electroneutral Na-Cl absorption (which represents the coupling of parallel ion exchanges—Na-H and Cl-HCO_3_) [8]. However, most critical to the development of ORS was that cholera enterotoxin (i.e., cyclic AMP) did not inhibit glucose-stimulated Na and thus fluid absorption (Fig. 1). Thus, the physiological basis of ORS rests on the demonstration that absorptive and secretory processes in the mammalian small intestine are separate and independent; that cholera-toxin-mediated cyclic AMP-induced active Cl secretion does not affect glucose-induced Na absorption; and conversely and most important, glucose stimulates Na absorption via a cyclic AMP-independent transport process (despite the stimulation of active Cl secretion by cyclic AMP) [7–10]. In addition, it was recently shown that glucose has an additional effect that will increase Na and fluid absorption: glucose reverses cyclic AMP’s down-regulation of Na-H exchanger 3 (NHE3) [11].

The third and most important event in the development and rapid acceptance of ORS in the treatment of acute diarrhea was its extensive and effective use under field conditions during the Bangladeshi war of independence in the early 1970s [12]. The effective deployment of ORS in refugee camps in Bengal demonstrated both its efficacy and effectiveness to provide rehydration during acute diarrheal illnesses that are self-limited (provided that the patient can be successfully rehydrated). During this period, with an extensive epidemic of cholera and other water-borne diarrheal illnesses, the effective use of ORS under field conditions established ORS as the mainstay of treatment for acute diarrhea. Indeed, many have concluded that ORS was the major therapeutic advance of the last (i.e., twentieth) century [13]!

## Modifications of WHO-ORS

Despite the great success of ORS in the treatment of acute infectious diarrhea over the ensuing quarter century, there have been several major efforts to modify the composition of ORS with the goal to improve its efficacy (as demonstrated in clinical trials) to reduce diarrhea (i.e., reduce both the time to first formed stool and stool volume) and its effectiveness (i.e., as established in field conditions). The use of one or more amino acids, disaccharides, and polymers (e.g., sucrose) added to ORS provided modest but not dramatic improvement in efficacy [14–19]. Major efforts have been made to employ food-based, cereal-based ORS formulations [20–25]. The initial ORS formulation (often referred to as WHO-ORS) is “isoosmolar” (i.e., 311 mOsm/kg H_2_0). Since food-based formulations result in hydrolysis of oligosaccharides and peptides in the proximal small intestine, resulting in the release of substantial amounts of amino acids and hexoses, these food-based ORS formulations have, in general, been hypo-osmolar (e.g., ~245 mOsm/kg H_2_0). Several appropriately designed randomized controlled trials have subsequently demonstrated that such formulations are significantly better than WHO-ORS (i.e., iso-osmolar) [23]. However, the question was raised whether the improved efficacy of meal-based ORS formulations was not due to the presence of food polymers per se but was a result of the hypo-osmolality of these formulations [26]. As a consequence, a series of studies were performed with hypo-osmolar ORS formulations comparing glucose and food-based compositions. These studies established the efficacy of hypo-osmolar, glucose-based formulations (without the presence of food polymers), which represented yet another milestone in the improvement of ORS [27•]. Since then, several governments in Asia and Africa have adopted the use of reduced osmolarity (or hypo-osmlar) ORS formulation as the standard ORS treatment for diarrhea [28].

Despite the ready demonstration that employment of ORS during episodes of acute diarrhea improves morbidity and mortality especially in young children, the actual usage of ORS has varied markedly over the past 30 years for many reasons, and remained relatively low and unchanged in many countries [29]. Early on, there were extensive media events promoting employment of ORS during episodes of acute diarrhea. These campaigns have been judged effective to increase ORS uptake, but have usually been intermittent in duration. Further, such efforts have frequently been superseded by maternal education programs that have focused on providing education addressing the totality of child welfare, with emphasis on breast feeding, vaccination programs, and other important health, nutrition, and hygiene issues for children, in addition to the employment of ORS, with consequent loss of focus on the latter. To maintain continued high levels of ORS, it is necessary to ensure continual media education. This need for continued and sustained education is critical, if only to provide sustained education of ORS for the women who become new mothers every year. Though deaths from diarrhea are decreasing, it is important to emphasize that acute diarrhea remains the second highest cause of mortality in children under the age of 5 in developing countries (and only slightly less than that of pneumonia) [30].

**Fig. 2:**
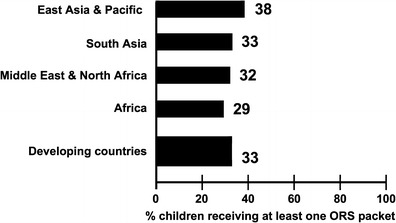
Percent of children under 5 with diarrhea receiving at least one ORS packet during illness, by region, 2005–2008. Adapted from the UNICEF-WHO publication, *Children are still dying from acute diarrhea*; *What can be done*? (2009) [29]

UNICEF/WHO released an important monograph in 2009 entitled *Diarrhoea*: *Why children are still dying and What can be done*? [31] Data presented in this publication emphasized that overall use of ORS by mothers in developing countries was only approximately 33 % (Fig. 2). This figure is far too low and certainly may be an important factor why children are still dying from episodes of acute diarrhea. An adequate explanation for this overall low use of ORS in the treatment of acute diarrhea is not totally known. In addition to cultural and access issues, an important issue is that ORS is not a drug (and hence at risk of not being perceived as a medicine of real value), nor is it expensive, and therefore may be considered as not as effective as treatments that are expensive and must be purchased from pharmacies (e.g., antibiotics). Though all hese reasons are distinct possibilities, we believe that a major contributing factor for ORS not being widely employed is its inability to reduce stool output dramatically. That is, mothers are most interested in relief of their child’s symptoms, i.e., reduction in diarrhea, and are not necessarily concerned about correction of acute dehydration and metabolic acidosis.

Thus, can ORS be improved so that its use will result in a substantial reduction in stool output, i.e., symptomatic relief of a child’s acute diarrhea? Since the small intestine is the site in which glucose stimulates active Na and fluid absorption (and glucose does not stimulate fluid and Na absorption in the colon), in 1993 we posed the question as to whether a new formulation could be developed that, in addition to using the small intestine, would also enhance Na and fluid absorption in the large intestine? Several observations make this therapeutic approach a very attractive concept; they emphasized the potential role of short-chain fatty acids (SCFA) and have been the focus of our studies for the past two decades [32, 33••, 34••, 35–37].

## Short-Chain Fatty Acids (SCFA)

**Fig. 3:**
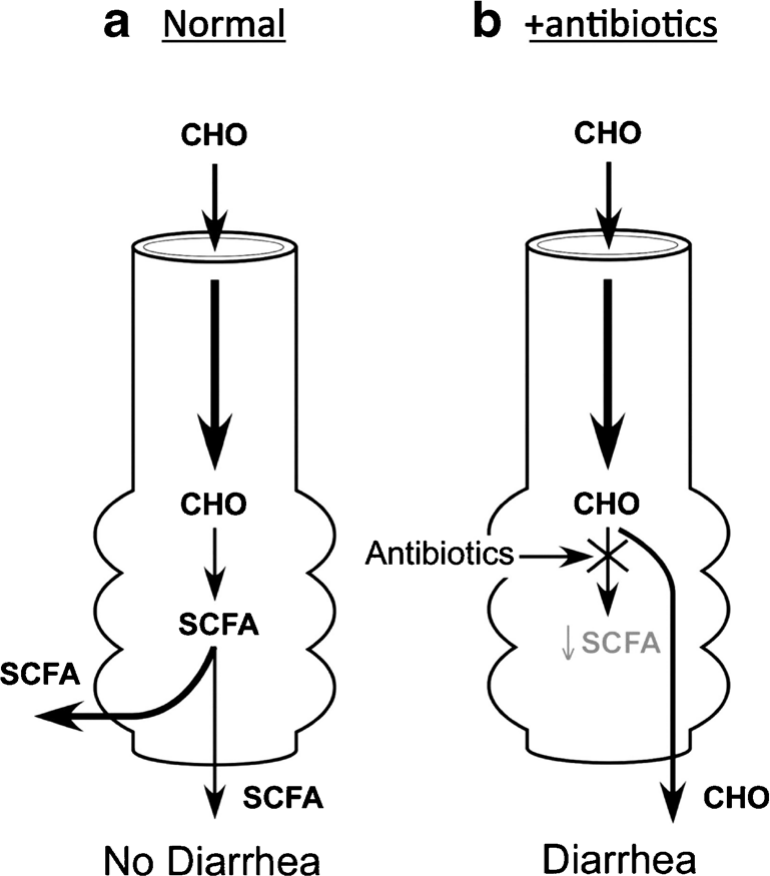
Importance of colonic microbiota in the production of short-chain fatty acids (SCFA) in the large intestine. **a** Carbohydrates that are not absorbed in the small intestine enter the colon where they are fermented to SCFA which are absorbed and stimulate Na and fluid absorption. SCFA production represents an adaptive process to conserve calories, Na and water. **b** Following antibiotic administration, there is a reduction in SCFA production often resulting in diarrhea as the non-absorbed carbohydrates induce fluid secretion via an osmotic mechanism. Much attention has focused on *C. difficile* in the genesis of antibiotic-associated diarrhea, but this represents no more than 20 % of all antibiotic-associated diarrhea, and evidence indicates that suppression of SCFA production likely is responsible for the majority of antibiotic-associated diarrhea

Interest in SCFA began approximately 30 years ago [38]. Studies at that time demonstrated that SCFA (primarily acetate, propionate, and butyrate) are the primary stool anion but are not present in the diet. These SCFA are synthesized (i.e., fermented) by colonic bacteria from non-absorbed carbohydrates (Fig. 3). Carbohydrates are not absorbed in the colon and small soluble carbohydrates induce fluid secretion on an osmotic basis; in contrast, SCFA are rapidly absorbed in the colon and stimulate fluid and Na absorption [39]. As a consequence, the production of SCFA represents an adaptive mechanism by which the colon conserves carbohydrate, calories, fluid, and electrolytes.

Studies were performed to identify the cellular mechanism by which SCFA are absorbed and stimulate Na absorption. These in vitro studies established a mechanism for butyrate absorption that is linked to both Na and Cl absorption [31, 40]. This model proposed that the uptake of butyrate across the apical membrane is a result of a butyrate–bicarbonate exchange that in turn is linked to a Cl–bicarbonate anion exchange and a Na–H exchange (NHE). To confirm the role of a Na–H exchange, additional experiments were performed in which Na was removed and 1 mM amiloride (which inhibits NHE3 function) was added, both of which inhibited butyrate-stimulation of Na absorption [31, 32]. As NHE3 is an apical Na–H exchange that is inhibited by cyclic AMP, the effect of cyclic AMP to alter butyrate-stimulation of Na absorption was also tested and surprisingly failed to alter Na absorption. Thus, in vitro butyrate stimulated Na absorption via a cyclic AMP-independent mechanism [32]. At approximately the same time, studies performed by B.S. Ramakrishna et al. in the in vivo ileum and colon established that SCFA stimulation of Na absorption was not altered by cholera toxin [33••]. Such an observation suggested that SCFA stimulated Na absorption via a cyclic AMP-independent process. As a consequence, these observations suggested that SCFA in the colon might function similar to glucose in the small intestine to enhance fluid and Na absorption during an acute diarrheal illness in which an enterotoxin, e.g., cholera enterotoxin or *E. coli* enterotoxins, has induced substantial losses of fluid and electrolytes via stimulation of active Cl/HCO3 secretion (and inhibition of Na–H exchange).

## Resistant Starch: A Delivery Mechanism for SCFA Production

How best to deliver SCFA to the colon represented a challenge. Feeding SCFA was not a possibility, as transport mechanisms had been identified in the small intestine for SCFA absorption [41, 42]. Thus, oral administration of SCFA would not result in the delivery of SCFA to the colon. Graeme Young suggested that use of resistant starch (RS), i.e., starch that was relatively resistant to amylase digestion in the small intestine [43], represented a potential approach to provide substrate for local intracolonic production of SCFA, as feeding of resistant starch to normal subjects had been shown to increase fecal butyrate excretion [44]. As a consequence, the initial critical experiment was to determine whether RS incubated in vitro with stool from patients with acute cholera would result in SCFA production [45]. Such in vitro experiments established that such stool (despite use of antibiotics in the treatment of cholera) possessed the ability to ferment RS with the production of SCFA including butyrate. This latter observation led to the initiation of three randomized, double-blind clinical trials that were performed at Christian Medical College (CMC)-Vellore in South India [46••, 47•, 48••].

## Clinical Trials with RS-ORS

**Fig. 4:**
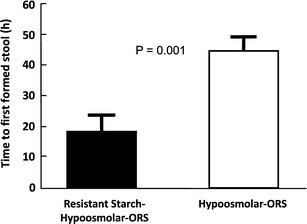
Time to the first formed stool. Results of a randomized, double-blind clinical trial in adults with acute cholera randomized to hypo-osmolar ORS or HAMS-hypo-osmolar ORS (shown here as resistant starch) [46••]. The resistant starch group had a 55 % reduction in time to first formed stool as well as reduction in stool output

These studies compared a specific RS (high-amylose maize starch, referred to as HAMS), hence HAMS–ORS, in two trials in adults with acute cholera and one in children with non-cholera diarrhea [46••, 47•, 48••]. One of the adult studies and the clinical trial in children used an iso-osmolar HAMS-ORS formulation, while the other adult study employed a hypo-osmolar HAMS-ORS formulation (Fig. 4). In all three studies, the HAMS-ORS formulation was associated with a 30–50 % reduction in time to the first formed stool—which represents a very significant reduction in stool output.

**Fig. 5:**
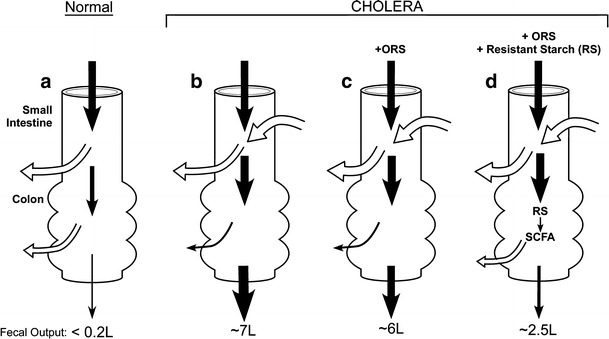
Model of intestinal fluid movement in health and in acute cholera treated with ORS. (*Width of arrows* provides approximation of fluid movement; values presented are per 24 h.) **a** In health, the major fraction of fluid entering the small intestine is absorbed with approximately 2 l reaching the colon where all but less than 200 ml is also absorbed.**b** In cholera, there is significant small intestinal fluid secretion secondary to active Cl secretion and inhibition of NaCl absorption (see Fig. 1) resulting in enhanced ileo-cecal flow; in addition, colonic Na-Cl and fluid absorption is reduced resulting in up to 7 l of diarrhea per day. **c** Administration of ORS does not alter active Cl secretion but enhances fluid absorption by stimulating active Na absorption (via a cAMP-independent process) in the small intestine correcting dehydration but only modestly reducing ileo-cecal flow. Colonic fluid absorption is not altered. **d** Administration of HAMS (a resistant starch)-ORS results in identical changes in the small intestine but in the colon HAMS is fermented to SCFA that stimulates Na absorption (also via a cAMP-independent process) resulting in further rehydration and a substantial reduction in diarrhea

Figure 5 summarizes the changes in small and large intestinal fluid movement in health (panel a), in patients with cholera (panel b) and then treated either with ORS (panel c) or with RS-ORS (panel d). Additional details are provided in the legend of Fig. 5.

## Toward Identification of the Optimal RS-ORS

These observations raised the important question whether HAMS-ORS would represent the ideal or optimal ORS to develop for world-wide adaption as the most efficacy ORS formulation. The answer to that query has not been resolved as yet, but preliminary studies suggest that other modified starches, especially those that qualify as resistant starch, might be equally or more efficacious. To address this possibility two linked experiments were designed: the initial one was an in vitro study in which 75 different compounds that had the potential for catabolism to yield SCFA were individually incubated with normal stool (unpublished observations). As some of these compounds were not starches, the term used to describe these compounds was *fermentable substances* (*FS*) or non-digestible carbohydrates. Six of these FS that yielded the highest concentration of SCFA during in vitro incubation with human stool were selected for in vivo studies in the rat using a newly developed experimental approach—a whole gut perfusion [49]. This experimental design was devised to reproduce acute diarrhea in humans by administering either enterotoxins of *Cholera vibrio* or *E. coli* to the rodent intestine that had been cannulated proximally at the pylorus and distally at the rectum. As a consequence, this experimental design permitted determination of fluid movement in the entire small and large intestine during perfusion with different electrolyte solutions. The results of these studies demonstrated that HAMS-ORS resulted in greater fluid absorption in the presence of cholera enterotoxin than the present standard ORS formulation—glucose–hypo-osmolar ORS. However, these experiments also revealed that two modified HAMS compounds (esterified starches containing varying amounts of acetyl groups) resulted in even higher rates of fluid absorption during cholera toxin-induced fluid secretion (unpublished observations).

In order to proceed to clinical trials with these esterified starches another problem required attention. Most of these starches when added to a glucose–electrolyte solution did not yield a clear solution but rather had an opaque appearance that, over a short period of time, often just 1 h, would settle out of suspension. As a result, efforts were required to identify one or more suspending agents that would ensure that, even without stirring, a child would receive the desired amount. These ORS formulations containing an anti-settling agent were also studied in the in vivo whole gut perfusion system. These studies demonstrated that there was no loss of the advantages provided by the esterified HAMS solutions with varying amounts of acetylation compared to the HAMS-ORS formulation (unpublished observations). As a consequence, clinical trials have been designed and recently started to determine which of these three HAMS-related ORS formulations each containing a suspending agent will result in greater efficacy in the treatment of acute diarrhea in adults. Such a compound will then be selected for more detailed studies including safety, efficacy in both adults and children, and, most importantly, in different areas of the developing world to establish its efficacy with several different pathogenic agents.

## Role of Zinc in ORS Therapy

Table 1 lists the composition of the present WHO-UNICEF recommended treatment for acute diarrhea. In addition to using a glucose–hypo-osmolar ORS formulation for rehydration, present guidelines also indicate that a 10-day course of Zn should be administered. This recommendation is based on several observations that demonstrated that Zn supplementation resulted in a 12–25 % reduction in acute diarrhea in children [50]. Total body stores of Zn in humans are normally quite low and diarrhea results in Zn losses. However, it has not been unequivocally established whether Zn supplementation in acute diarrhea is efficacious only in Zn-deficient children or in both Zn-deficient and Zn-surfeit children. If Zn treatment reduces diarrhea only in Zn-deficient children, then the mechanism of Zn’s action would likely represent correction of a Zn micro-nutrient deficiency. In support of this possibility are experimental observations that small intestinal function is abnormal in Zn-deficiency and that intestinal fluid secretion is both enhanced in Zn-deficiency and corrected by Zn administration [51, 52]. In contrast, if Zn treatment of diarrhea is also effective in Zn-surfeit children (as well as in Zn-deficient children), correction of a Zn micro-nutrient deficiency would not be an adequate explanation for both observations. It would then be necessary to postulate that Zn would also be efficacious as an anti-diarrheal agent, i.e., that Zn could enhance fluid and Na absorption and/or inhibition fluid and Cl secretion. Indeed, recent studies have demonstrated that Zn can enhance Na absorption via virtue of stimulation of NHE3 function [53] and can inhibit cyclic AMP-induced Cl secretion by functioning as a basolateral K channel blocker [54].

**Table 1:** Composition of presently WHO-UNICEF recommended oral rehydration solution

Glucose	75 mM
Sodium	75 mM
Chloride	65 mM
Potassium	20 mM
Citrate	10 mM
Total osmolarity	245 mOsm/Kg H_2_O

## Conclusions

Oral rehydration therapy (ORT) has been developed over the past 40 years and has been established as the standard of therapy for the treatment of the dehydration and metabolic acidosis associated with acute diarrhea. The use of ORS has been attributed as the primary reason for the substantial reduction in morbidity and mortality of acute infectious diarrhea. Despite these successes, ORS is not employed by mothers to the extent that one would anticipate, and multiple efforts have been made to improve the formulation of ORS. This review describes our efforts to establish the incorporation of a fermentable (or resistant) starch into ORS based on (1) the delivery of such a fermentable starch to the colon where fermentation to SCFA occurs, and (2) SCFA stimulation of colonic Na absorption via a cyclic AMP-independent mechanism. As a result, these newly formulated ORS that are presently being studied in clinical trials represent a “dual-action” ORS in which Na and fluid absorption is enhanced in both small and large intestine.
